# Modeling growth and development of hydroponically grown dill, parsley, and watercress in response to photosynthetic daily light integral and mean daily temperature

**DOI:** 10.1371/journal.pone.0248662

**Published:** 2021-03-25

**Authors:** Kellie J. Walters, Roberto G. Lopez

**Affiliations:** 1 Department of Horticulture, Michigan State University, East Lansing, Michigan, United States of America; 2 Plant Sciences Department, University of Tennessee, Knoxville, Tennessee, United States of America; Lovely Professional University, INDIA

## Abstract

In controlled environments, crop models that incorporate environmental factors can be developed to optimize growth and development as well as conduct cost and/or resource use benefit analyses. The overall objective of this study was to model growth and development of dill ‘Bouquet’ (*Anethum graveolens*), parsley ‘Giant of Italy’ (*Petroselinum crispum*), and watercress (*Nasturtium officinale*) in response to photosynthetic daily light integral (DLI) and mean daily temperature (MDT). Plants were grown hydroponically in five greenhouse compartments with MDTs ranging from 9.7 to 27.2 °C under 0%, 30%, or 50% shade cloth to create DLIs ranging from 6.2 to 16.9 mol·m^‒2^·d^‒1^. MDT and DLI interacted to influence dill fresh mass and height, and watercress maximum quantum yield of dark adapted leaves (F_v_/F_m_), height, and branch number while only MDT affected dill leaf number and watercress fresh mass and branch length. Besides dry matter concentration (DMC), parsley was influenced by MDT and not DLI. Increasing MDT from ≈10 to 22.4 °C (parsley) or 27.2 °C (dill and watercress), linearly or near-linearly increased fresh mass. For dill, increasing DLI decreased fresh mass when MDT was low (9.7 to 13.9 °C) and increased fresh mass when MDT was high (18.4 to 27.2 °C). DMC of dill, parsley, and watercress increased as MDT decreased or DLI increased, indicating a higher proportion of plant fresh mass is water at higher MDTs or lower DLIs. With these data we have created growth and development models for culinary herbs to aid in predicting responses to DLI and MDT.

## Introduction

The fresh culinary herb market in the United States (U.S.) is at the introductory stage of its product life cycle, with growth of 10% to 14% per year from 2004 to 2014 [1]. In 2014, 1.3 million m^2^ of controlled environment production yielded over 16.1 million kg of fresh-cut herbs (3.5 million kg from hydroponic systems) and total producer sales of $71 million [2]. However, greenhouse herb growers face multiple challenges that impede their full production potential. From a recent survey of U.S. hydroponic growers, Walters et al. [3] reported that the creation of “production recipes” taking multiple environmental parameters into account would be one of the most beneficial research topics to the industry.

Plant growth, development, and quality are primarily influenced by light (radiant energy) and temperature (thermal energy). Photosynthesis and thus growth, biomass accumulation, crop quality, and yield are primarily influenced by the photosynthetic daily light integral (DLI; mol·m^‒2^·d^‒1^). In commercial greenhouse and indoor plant production, one main limitation to high rates of photosynthesis is low DLI. In the northern U.S., average outdoor DLI in December and January often falls below 10 mol·m^‒2^·d^‒1^, the threshold below which the growth of most floriculture crops is unacceptable [4], and greenhouse superstructure can further reduce DLI at the plant canopy by 35% to 70% [5]. It is well established that increasing the cumulative radiation intensity to a crop-specific optimum increases photosynthesis, biomass, and overall plant quality; thus, low DLIs during winter months can reduce plant quality [6, 7]. Recent research has determined that increasing DLI from 2 to 19 mol·m^‒2^·d^‒1^ [parsley ‘Giant of Italy’ (*Petroselinum crispum*)] or 20 mol·m^‒2^·d^‒1^ [dill ‘Fernleaf’ (*Anethum graveolens*)] increased fresh mass and harvestable yield [7]. However, to increase fresh mass yield during low solar radiation conditions in a greenhouse or indoors, supplemental or sole-source lighting is required, respectively.

Temperature is the primary determinant of developmental rates, including the progress to flower and leaf unfolding rate, but it also plays a role in growth and yield [8–11]. More precisely, it is temperature integrated over time, typically a 24-h period, referred to as mean daily temperature (MDT). A temperature response curve can describe the relationship between development and MDT. Below the base temperature (T_b_), development ceases. As MDT increases between T_b_ and the optimum temperature (T_opt_), development increases at a (near-) linear rate. At T_opt_, the rate of development is at its maximum. Temperature-dependent increases in photosynthesis, growth, and development in the linear range between T_b_ and T_opt_ are largely due to increased enzymatic activity [12]. As MDT increases beyond T_opt_, the rate of growth and development decreases until the maximum temperature (T_max_), above which it ceases.

Many researchers have demonstrated that plant development in response to temperature is integrated over one day. Time to flower of African violet ‘Utah’ (*Saintpaulia ionantha*) was similar whether the day temperature was less than, the same as, or greater than the night temperature as long as MDT was the same [13]. Similarly, Hurd and Graves [14] determined that differences in day and night temperature did not influence time to flower or yield of tomato ‘Marathon’ (*Solanum lycopersicum*) as long as MDT was the same and the MDT was between T_b_ and T_opt_. Tomato ‘Counter’ truss and leaf number and early yields depended on MDT, regardless of night temperature, although later yields were higher when night temperature was higher at the same MDT [15].

Plant response to temperature can also be integrated over temporal durations longer than one day. De Koning [16] determined that tomato ‘Counter’ can integrate temperatures over a 12 day period as long as the temperature fluctuation over the integration period was less than 6 °C, and Körner and Challa [17] determined that chrysanthemum ‘Reagan Improved’ (*Chrysanthemum morifolium*) development can be integrated based on temperatures over a six day period. Chang et al. [18] found that fresh mass of sweet basil ‘Sweet Genovese’ (*Ocimum basilicum*) did not differ when grown in six different temperature treatments, consisting of various combinations of one week each at 15, 25, or 30 °C, but the same average temperature over a three week period.

Radiation and temperature can interact to affect plant growth and development. Metabolic processes such as photosynthesis increase as temperatures increases to a process-specific T_opt_. Given this, both radiation and temperature impact photosynthesis. Additionally, dark respiration rates are affected by both night temperature and day radiation intensity. Respiration rate increases as temperature increases, reducing overall net carbon dioxide (CO_2_) assimilation [19, 20]. Additionally, but to a smaller extent, increasing day radiation intensity increases dark respiration [20].

In both acclimated and non-acclimated plants; photosynthetic T_opt_ generally increases as radiation intensity increases [8, 20]. Increasing DLI from ~3 to ~13 mol·m^‒2^·d^‒1^ increased the photosynthetic T_opt_ in both light-treatment acclimated and non-acclimated cucumber ‘Moskovsky Teplichnyi’ (*Cucumis* s*ativus*) seedlings [8]. In non-experimental condition acclimated carnation ‘Cerise Royalette’ (*Dianthus caryophyllus*), the T_opt_ for whole-plant CO_2_ assimilation increased as radiation intensity increased [20]. The CO_2_ assimilation T_opt_ was between 5 and 10 °C when radiation intensity was 205 μmol·m^–2^·s^–1^ but increased to 27 °C when radiation intensity was 2,050 μmol·m^–2^·s^–1^.

Models to predict growth and developmental parameters based on DLI and MDT in combination have been generated for economically important floriculture crops including petunia ‘Easy Wave Coral Reef’ and ‘Wave Purple’ (*Petunia ×hybrida* [21]), celosia ‘Gloria Mix’ (*Celosia argentea*), impatiens ‘Accent Red’ (*Impatiens walleriana* [22]), salvia ‘Vista Red’ (*Salvia splendens*), marigold ‘Bonanza Yellow’ (*Tagetes patula* [23]), cyclamen ‘Metis Scarlet Red’ (*Cyclamen persicum* [24]), and pansy ‘Universal Violet’ (*Viola* ×*wittrockiana* [25, 26]). Although MDT and DLI individually have been modeled in food crops mentioned previously, concurrent temperature and radiation-dependent modeling in food and agronomic crops has been more limited; models have been generated to predict the photosynthetic optimum of young cucumber ‘Moskovsky Teplichnyi’[8] and white clover (*Trifolium repens*) [19]. To our knowledge, the influence of MDT and DLI on culinary herbs has yet to be published. Understanding this interaction will allow us to develop more refined crop production models and provide recommendations to improve yields, improve grower profitability, and reduce excess energy costs.

Although technology to manipulate the greenhouse temperature and DLI exists, its utility is limited when responses to both DLI and MDT are largely unknown for many crops. Hence, models to predict growth and development are integral to optimizing culinary herb production and conducting cost and/or resource use benefit analyses in response to environmental changes. Therefore, the overall objectives of this study were to determine the extent DLI and MDT influence the growth and development of culinary herbs and greens that can be readily produced in hydroponic production including dill, parsley, and watercress (*Nasturtium officinale*), and to create quantitative models to predict crop growth and development. Our hypothesis was that DLI would interact with MDT and the T_opt_ of each species would increase as DLI increased.

## Results

### Dill

**Fig 1:**
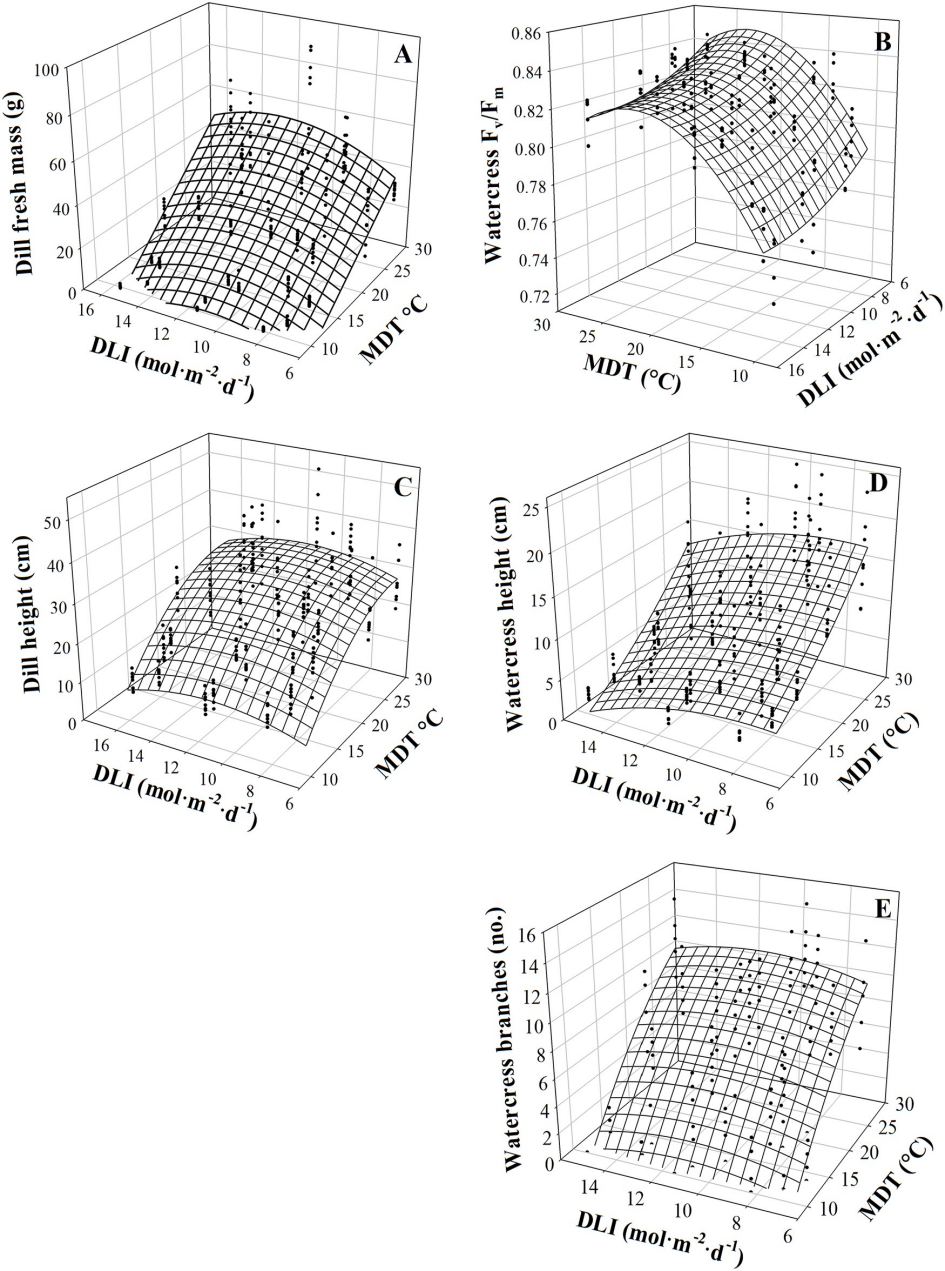
Mean daily temperature (MDT) and daily light integral (DLI) effects on dill (*Anethum graveolens*) fresh mass (A) and height (C), watercress (*Nasturtium officinale*) maximum quantum yield of dark-adapted leaves (F_v_/F_m_; B), height (D), and branch number (E). Response surfaces represent model predictions. Coefficients for these models are presented in Table 1. Models are each based on 270 individual measurements.

MDT and DLI interacted to influence dill fresh mass (Table 1, Fig 1A). Within the observed ranges of MDT and DLI, MDT had a qualitatively larger effect than DLI. As MDT increased from 11.4 to 26.9 °C when DLI was low (7.5 to 8.7 mol·m^‒2^·d^‒1^), fresh mass increased 11.7-fold (by 43.4 g). Increasing DLI had a negative effect on fresh mass when MDT was low (9.7 to 13.9 °C) and a positive effect when MDT was high (18.4 to 27.2 °C). For example, when grown at 9.7 or 13.9 °C, increasing DLI from ~8.0 to ~15.5 mol·m^‒2^·d^‒1^, resulted in 25% (0.6 g) and 53% (6.6 g) less fresh mass, respectively. In contrast, increasing DLI from ~6 to ~15 mol·m^‒2^·d^‒1^, increased fresh mass 57% (19 g) at 27.2 °C. Height at harvest followed a similar trend but increased over two-fold (>15.8 cm) as MDT increased from 9.7 to 21.6 °C, then plateaued (Table 1, Fig 1C).

**Table 1:** Regression analysis parameters, R^2^ or r^2^, and calculated base temperatures (T_b_) or equation required to calculate T_b_ for dill (*Anethum graveolens*), parsley (*Petroselinum crispum*), and/or watercress (*Nasturtium officinale*) fresh mass, leaf number, height, Dry Matter Concentration (DMC), maximum quantum yield of dark-adapted leaves (F_v_/F_m_), branch number, and branch length in response to mean daily temperature (MDT; °C) and daily light integral (DLI; mol·m^‒2^·d^‒1^).

Parameter	y0	(a) MDT	(b) DLI	(c) MDT^2^	(d) DLI^2^	(e) MDT*DLI	R^2^ or r^2^	T_b_
Dill ‘Bouquet’								
Fresh mass (g)	-78.53 ^z^	2.33	9.88		-4.80 E-1	6.98 E-2	0.775	28.14–3.48*DLI + 0.156*DLI^2^ ^y^
	(14.29) ^x^	(4.08 E-1)	(2.22)		(8.63 E-2)	(3.63 E-2)		
Leaf no.	-2.41	3.67 E-1					0.681	6.6
	(0.94)	(4.91 E-2)						
Height (cm)	-25.11	3.40	1.88	-7.54 E-2	-1.28 E-1	5.64 E-2	0.599	-
	(9.47)	(5.58 E-1)	(1.36)	(1.29 E-2)	(5.32 E-2)	(2.23 E-2)		
DMC (g·kg^‒1^)	222.38	-10.06		1.90 E-1			0.742	-
	(25.50)	(2.90)		(7.72 E-2)				
DMC (g·kg^‒1^)	75.63		2.98				0.168	-
	(13.63)		(1.20)					
Parsley ‘Giant of Italy’								
Fresh mass (g)	-55.16	6.50		-1.16 E-1			0.642	10.4
	(26.30)	(3.02)		(8.03 E-2)				
Height (cm)	-32.65	4.65		-9.01 E-2			0.780	-
	(6.79)	(7.82 E-1)		(2.08 E-2)				
Leaf no.	-2.56	3.62 E-1					0.693	7.1
	(6.70 E-1)	(3.50 E-2)						
F_v_/F_m_ ^w^	-3.24 E-2	8.69 E-1		2.18 E-1			0.340	-
	(1.79)	(1.77)		(2.09 E-1)				
DMC (g·kg^‒1^)	163.15	-1.11					0.109	-
	(9.75)	(5.10 E-1)						
DMC (g·kg^‒1^)	115.69		2.37				0.152	-
	(8.09)		(6.76 E-1)					
Watercress								
Height (cm)	-12.46	7.14 E-1	1.98	1.25 E-2	-8.00 E-2	-3.95 E-2	0.722	-
	(4.50)	(2.93 E-1)	(6.70 E-1)	(6.7 E-3)	(2.84 E-2)	(1.16 E-2)		
Branch no.	-15.47	1.10	9.49 E-1	-1.57 E-2	-5.28 E-2	1.41 E-2	0.808	16.72–1.20*DLI + 0.0571*DLI^2^ ^y^
	(2.84)	(1.84 E-1)	(4.22 E-1)	(4.2 E-3)	(1.79 E-2)	(7.3 E-3)		
F_v_/F_m_	7.12 E-1	1.45 E-2	-6.40 E-3	-4. E-4		2. E-4	0.554	-
	(3.04 E-2)	(2.60 E-3)	(2.0 E-3)	(5.9 E-5)		(1. E-4)		
Fresh mass (g)	-12.03	1.14					0.691	10.6
	(1.52)	(7.89 E-2)						
Branch length (cm)	-15.14	1.31					0.850	11.6
	(1.71)	(8.88 E-1)						
DMC (g·kg^‒1^)	116.23	-1.88					0.274	-
	(11.78)	(6.12 E-1)						
DMC (g·kg^‒1^)	58.39		2.15				0.077	-
	(12.93)		(1.16)					

^z^ Coefficients for model equations were used to generate Figs 1–3.

^y^ T_b_ changes based on DLI.

^x^ Standard error (se).

^w^ Exponential rise to a maximum model used: *f* = y0 + a*(1-exp(-c*MDT)).

Unless noted, all models are in the form of: *f* = y0 + a*MDT + b*DLI + c*MDT^2^ + d*DLI^2^ + e*MDT*DLI.

**Fig 2:**
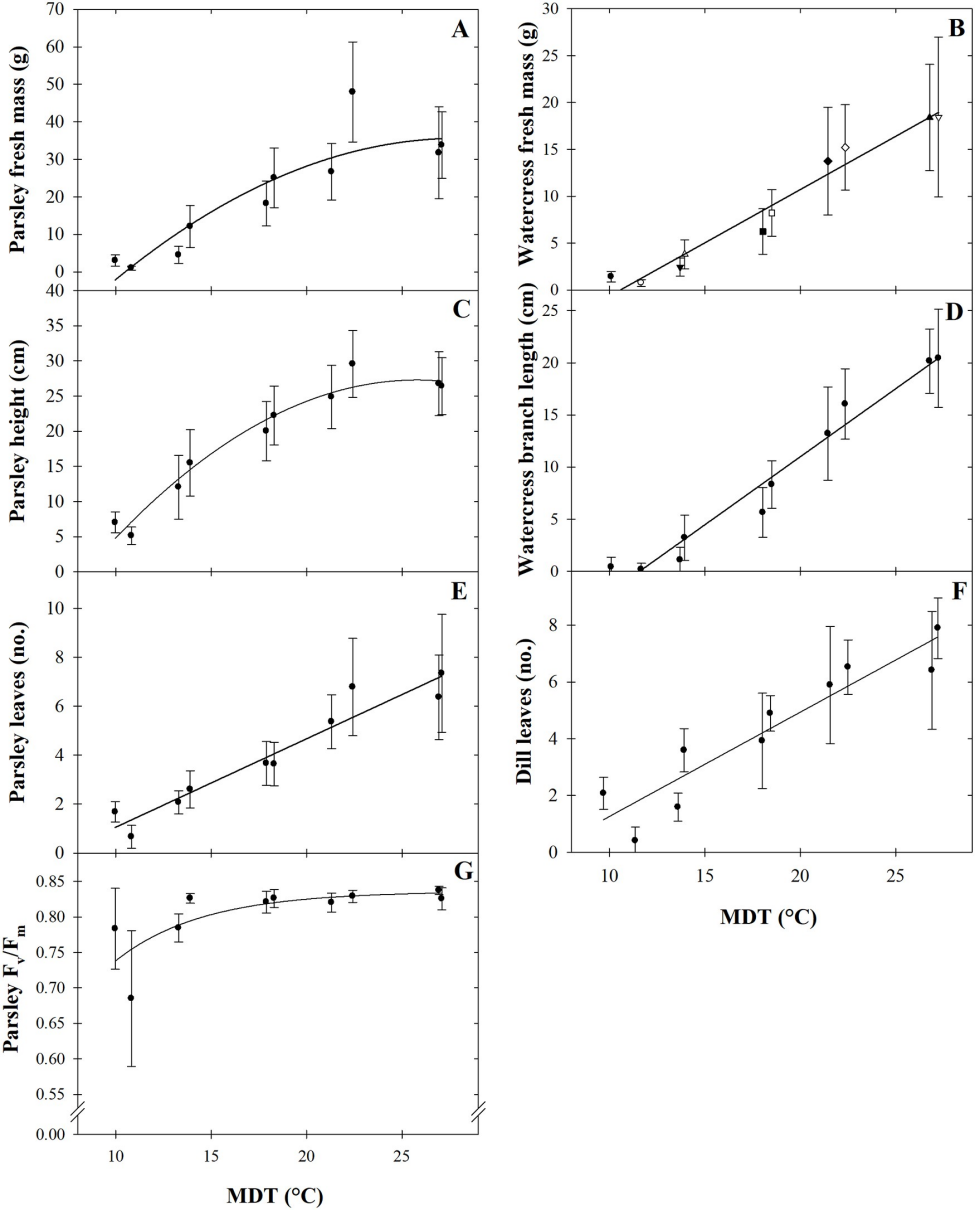
Mean daily temperature (MDT) effects on parsley (*Petroselinum crispum*) fresh mass (A), height (C), leaf number (E), and maximum quantum yield of dark-adapted leaves (F_v_/F_m_; G), watercress (*Nasturtium officinale*) fresh mass (B) and branch length (D), and dill (*Anethum graveolens*) leaf number (F). Lines represent model predictions, with the coefficients for these models presented in Table 1. Symbols (means ± sd) represent measured data (n = 27).

**Fig 3:**
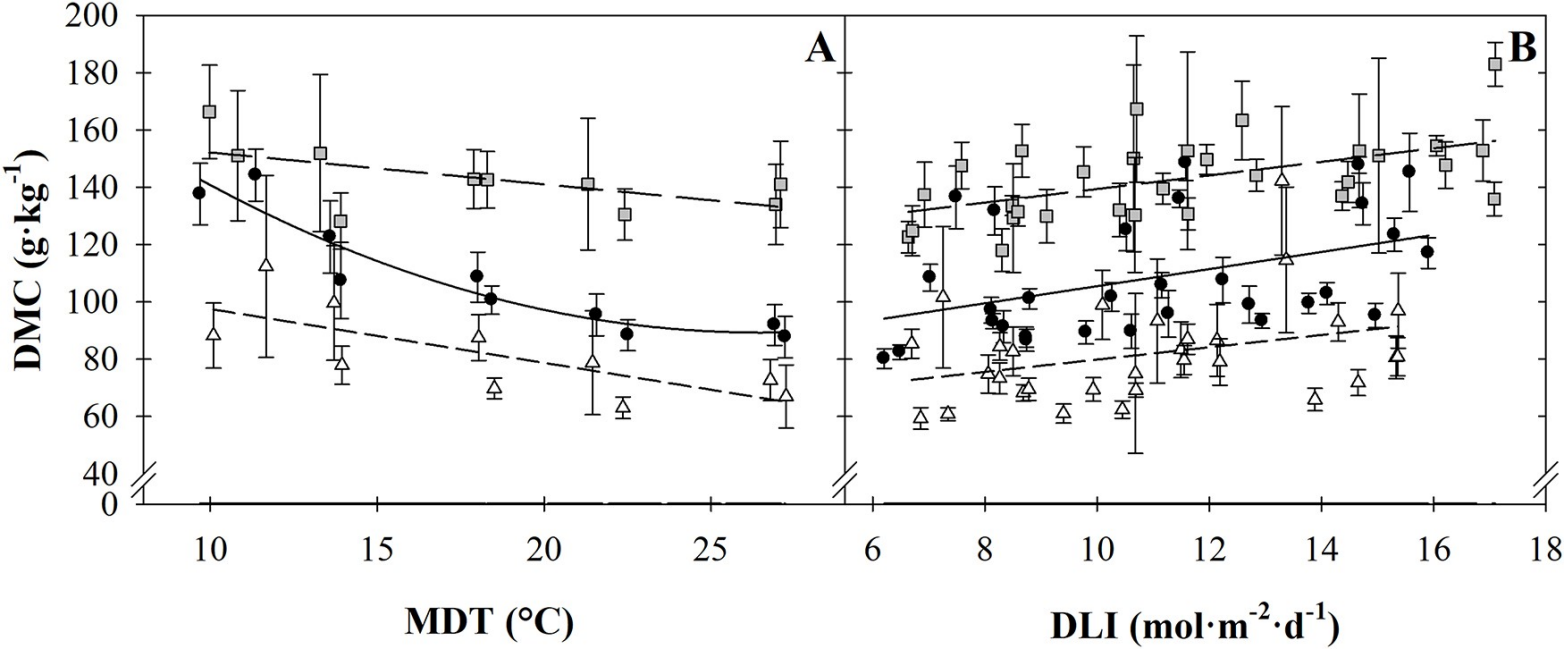
Mean daily temperature (MDT; A) and daily light integral (DLI; B) effects on dill (*Anethum graveolens*; ●), parsley (*Petroselinum crispum*; grey box, and watercress (*Nasturtium officinale*; Δ) dry matter concentration (DMC). Lines represent model predictions, with the coefficients for these models presented in Table 1. Symbols (means ± sd) represent measured data used to generate the models (A, n = 27; B, n = 9).

The number of unfolded leaves increased linearly as MDT increased (Table 1, Fig 2F). Six more leaves unfolded when MDT increased from 13.6 to 27.2 °C. Dry matter concentration (DMC) of dill increased as MDT decreased or DLI increased (Table 1, Fig 3A and 3B). As MDT increased from 9.7 to 27.2 °C, DMC decreased by an average of 37% (53 g·kg^‒1^). As DLI increased by 4.3 to 8.4 mol·m^‒2^·d^‒1^, DMC increased by 12% (12 g·kg^‒1^).

### Parsley

Parsley DMC, fresh mass, maximum quantum yield of dark-adapted leaves (F_v_/F_m_), height, and leaf number were influenced by MDT, and DMC was also influenced by DLI. As MDT increased from 10.0 to 22.4 °C, parsley fresh mass, height, and leaf number increased 13-fold (45 g), 3-fold (22.6 cm), and 1.3-fold (5 leaves), respectively (Table 1, Fig 2A, 2C and 2E). As MDT further increased from 22.4 to 27.1 °C, fresh mass and height decreased by 29% (14 g) and 11% (3.2 cm), respectively, and leaf number did not increase. F_v_/F_m_ was similar among plants grown at MDTs of 13.9 to 27.1 °C. However, F_v_/F_m_ declined at MDTs below 13.9 °C (Fig 2G).

Similar to dill, DMC of parsley increased as MDT decreased or DLI increased (Fig 3A and 3B). DMC was an average of 12% (19 g·kg^‒1^) lower as MDT increased from 10.0 to 27.1 °C, and 11% (14 g·kg^‒1^) lower as DLI increased by 4.3 to 10.2 mol·m^‒2^·d^‒1^.

### Watercress

MDT but not DLI influenced watercress fresh mass and branch length (Table 1, Fig 2B and 2D). As MDT increased from 10.1 to 27.2 °C, fresh mass and branch length increased by 50-fold (20 g) and 11-fold (17 cm), respectively.

MDT and DLI interacted to influence the height of watercress at harvest. Plants were taller as MDT increased (Table 1, Fig 1D). The effect of MDT was linear; increasing MDT from 11.7 to 26.8 °C increased height 3-fold (15.1 cm) at a DLI of ~12 mol·m^‒2^·d^‒1^. The relationship between height and DLI was quadratic. For instance, at a MDT of 26.8 °C, decreasing DLI from 12.2 to 9.9 mol·m^‒2^·d^‒1^ increased height 17% (3.4 cm) while further decreasing DLI to 8.8 mol·m^‒2^·d^‒1^ did not further change height.

Watercress F_v_/F_m_ was influenced by an interaction of MDT and DLI (Table 1, Fig 1B). MDT had a larger influence than DLI; F_v_/F_m_ values were similar between plants grown from 13.9 to 27.2 °C, but F_v_/F_m_ values decreased as MDT decreased below 13.9 °C. The effect of DLI was MDT-dependent. Decreasing DLI when MDT was high (13.9 to 27.2 °C) had little effect on F_v_/F_m_ (0.00 to 0.02 F_v_/F_m_ increase) while decreasing DLI when MDT was low (10.1 to 13.7 °C) resulted in greater F_v_/F_m_ increases (0.04 to 0.05). Watercress branch number was also influenced by the interaction of MDT and DLI; increasing MDT increased branch number to a greater extent than increasing DLI (Table 1, Fig 1E).

Similar to dill and parsley, DMC of watercress was influenced individually by both MDT and DLI. DMC increased as MDT decreased or DLI increased (Table 1, Fig 3A and 3B).

## Discussion

### Photosynthetic DLI

The large role photosynthetic DLI plays in biomass accumulation and thus fresh yield has been well documented in food and ornamental crops [4, 6, 7, 27, 28]. In our experiment, the presence and extent of positive DLI effects were MDT dependent (Table 1). Additionally, in some cases, DLI did not influence the growth nor developmental parameters measured. Though less frequently published, research has reported little to no differences in shoot biomass due to increasing radiation intensity. For example, Liu and Su [29] reported no difference in shoot dry biomass between *Taxus* grown under full sunlight, 40% to 60% full sunlight, and <10% full sunlight. Similarly, increasing the DLI from ~5 to ~20 mol·m^‒2^·d^‒1^ for gaura ‘Siskiyou Pink’ (*Gaura lindheimeri*) and ~6 to ~12 mol·m^‒2^·d^‒1^ for angelonia ‘AngelMist White Cloud’ (*Angelonia angustifolia*) liners resulted in minimal to no dry mass increase [30, 31].

While DLI did not affect parsley in our study (besides DMC), Litvin-Zabal [7] reported that increasing DLI from 2 to 19 mol·m^‒2^·d^‒1^ increased fresh mass 4-fold (77 g) after four weeks at 22.7 °C MDT when three plants were grown per cell. Additionally, researchers have reported that increasing DLI from 7 to 18 mol·m^‒2^·d^‒1^ increased parsley ‘Giant of Italy’ fresh mass by 120% (13.3 g) four weeks after transplant [32]. The lack of a DLI effect in our study may be due to low plant density reducing radiation interception competition or a long propagation time (five weeks) relative to finishing time (four weeks) where the treatments were applied.

The influence of DLI on plant height and branch number at harvest without affecting fresh mass may be partially explained by DMC. An increase in DMC as DLI increases has been documented across many crops [6, 7, 33]. Increasing DLI, to a certain extent, increases photosynthesis and carbon fixation, which increases carbohydrate accumulation and is reflected in a higher dry mass. In the case of watercress, although fresh mass was not affected by DLI, plants grown under higher DLIs had higher carbon fixation and higher dry masses, thus, higher DMC.

### Temperature

Models to predict plant growth and development can be generated based on temperature response curves. Karlsson et al. [10] developed a model to predict leaf unfolding rates of hibiscus ‘Brilliant Red’ and ‘Pink Versicolor’ (*Hibiscus rosa-sinensis*). Since the rate of development between T_b_ and T_opt_ increases linearly as temperature increases, their models were most accurate when temperatures were within the linear range and plants were vegetative. In our study, parsley fresh mass increased as MDT increased from ~10.0 to 22.4 °C, while dill and watercress fresh mass increased from ~10.0 to 27.2 °C (Table 1, Figs 1A, 2A and 2B). By fitting a quadratic curve for parsley, a T_opt_ of 28 °C for fresh mass was calculated. The T_opt_ for dill and watercress fresh mass would be >27.2 °C, since they had a linear response and supraoptimal temperatures were not attained. Our estimation is higher than Currey et al. [34], who calculated parsley ‘Giant of Italy’ and dill ‘Fernleaf’ had a fresh mass T_opt_ of 22.9 and 22.5 °C, respectively. We hypothesize the T_opt_ differences between studies can be attributed to several factors including container versus hydroponic production, different cultivars (dill), and a higher DLI (19.5 mol·m^‒2^·d^‒1^) in their study.

Fraszczak and Knaflewski [9] grew dill and parsley at MDTs of ~13, ~18, and ~23 °C, and DLIs of 2.9 and 3.8 mol·m^‒2^·d^‒1^. Fresh mass of dill was responsive to temperature and increased from 5.0 to 6.5 g as temperature increased, whereas parsley fresh mass was similar when grown at ~13 and ~23 °C. However, the DLIs utilized in this experiment were very low and unsuitable for commercial production. Though our regression models do not include data below 6.2 mol·m^‒2^·d^‒1^, their results concur with our findings for dill that when DLI was relatively low (7.5 to 8.7 mol·m^‒2^·d^‒1^), increasing MDT increased fresh mass. Additionally, the fact that all three species had lower DMC with increased MDT may be explained by increased respiratory activity and reduced carbon fixation.

While watercress grows in cool streams across the U.S. [35], high air temperatures in our study did not negatively influence growth since fresh mass increased as MDT increased from 10.0 to 27.2 °C (Table 1, Fig 2B). Likewise, Engelin-Eigles et al. [36] found that increasing MDT from 10 to 20 °C increased fresh mass by ~50 g two weeks after transplant; however, they found that fresh mass was similar between 20 and 25 °C.

When determining planting density, branch length can be taken into account if overlapping branches are undesirable due to harvesting and automation. Based on our results, to avoid overlapping branches when harvested after two weeks, watercress should be grown at least 4 cm apart when MDT is 13 °C and 40 cm apart when MDT is 26 °C, while DLI did not influence branch length (Table 1).

### DLI and MDT interactions

The interaction of DLI and MDT was species- and parameter-dependent; it did not influence any parsley growth or developmental parameters measured but did influence all measured dill parameters except for DMC and leaf number. Additionally, watercress height, branch number, and F_v_/F_m_ were influenced by the interaction of DLI and MDT, but fresh mass, branch length, and DMC were not (Figs 1–3). In other studies, MDT and DLI interacted to influence rate of development (i.e. time to flower), flower number, height, and dry weight of salvia ‘Vista Red’, but only time to flower of marigold ‘Bonanza Yellow’ and impatiens ‘Accent Red’, and height and flower number of celosia ‘Gloria Mix’ [22, 23].

In general, increasing MDT has a greater positive effect on growth and photosynthesis when radiation intensity is higher [8, 19]. In our study, the influence of MDT on dill fresh mass was DLI dependent; increasing DLI had a small negative effect when MDT was low (9.7 to 13.9 °C) but a positive effect when MDT was high (18.4 to 27.2 °C; Table 1, Fig 1). These results agree with Litvin-Zabal [7] who reported that at a MDT of 22.7 °C, increasing the DLI from 2 to 20 mol·m^‒2^·d^‒1^ increased dill fresh mass. However, based on our results, increasing dill fresh mass when MDT is 15 °C or below will require reducing the DLI to 11.4 mol·m^‒2^·d^‒1^ or below to achieve maximum fresh yield. Similar detrimental effects of increased radiation intensity at low temperatures have been documented; at 10 °C, increasing the DLI from 10 to 33 mol·m^‒2^·d^‒1^ negatively affected sorghum (*Sorghum bicolor*) photosynthetic rate and appearance [37]. Additionally, in cucumber ‘Moskovsky Teplichnyi’ grown under varying radiation intensities and temperatures, researchers found the effect of temperature on CO_2_ assimilation was attenuated when the growing environment DLI was lower (~3 mol·m^‒2^·d^‒1^ compared to ~13 mol·m^‒2^·d^‒1^) [8]. Conversely, in celosia ‘Gloria Mix’, a greater increase in height at flowering occurred with increasing MDT when DLI was low, and the MDT-dependent increase in height was attenuated at higher DLIs [22]. When non-light treatment acclimated white clover was grown at 10 °C, increasing radiation intensity from 170 to 900 μmol·m^–2^·s^–1^ did not influence CO_2_ uptake; however, increasing the growing temperature to 30 °C increased CO_2_ uptake with increased radiation intensity [19]. Similarly, in non-CO_2_, -MDT, or -radiation intensity acclimated carnations ‘Cerise Royalette’, when the CO_2_ concentration was 700 mg·L^–1^, increasing MDT had little effect on CO_2_ uptake when radiation intensity was 205 μmol·m^–2^·s^–1^ but a large effect when radiation intensity was 2,050 μmol·m^–2^·s^–1^ [20]. Similarly, in the current study, increasing MDT from 11.4 to 27.2 °C increased fresh mass by 52.9 g when dill was grown under a DLI of ~15 mol·m^‒2^·d^‒1^, but only by 45.6 g when grown under ~8 mol·m^‒2^·d^‒1^ (Fig 1A). While some studies grow plants in a common environment (non-acclimated plants) to generate radiation intensity and temperature photosynthetic response models, these models are only accurate for the environmental condition they are acclimated to [8]. However, general trends can be drawn in parallel to acclimated responses. Optimal environmental parameters, T_opt_ for example, can change depending on the growing environment [8, 20]. For example, in cucumber ‘Moskovsky Teplichnyi’, a 5 °C increase in growing temperature resulted in a 1 °C increase in photosynthetic (CO_2_ assimilation) T_opt_ [8].

### Modeling

Identifying optimal conditions based on other environmental parameters is integral to improving production efficiencies to achieve the producer- and situation-dependent desired outcome, whether it is high biomass, compact plants, more leaves, a higher DMC, or parameters not included in this study including high phytonutrient concentrations and a longer postharvest life [3]. For example, when DLI cannot be significantly altered but MDT can, increasing MDT is a useful strategy to increase fresh mass. However, beyond the optimal MDT, not only will yield be reduced, but excess resources will be spent heating the growing environment.

**Fig 4:**
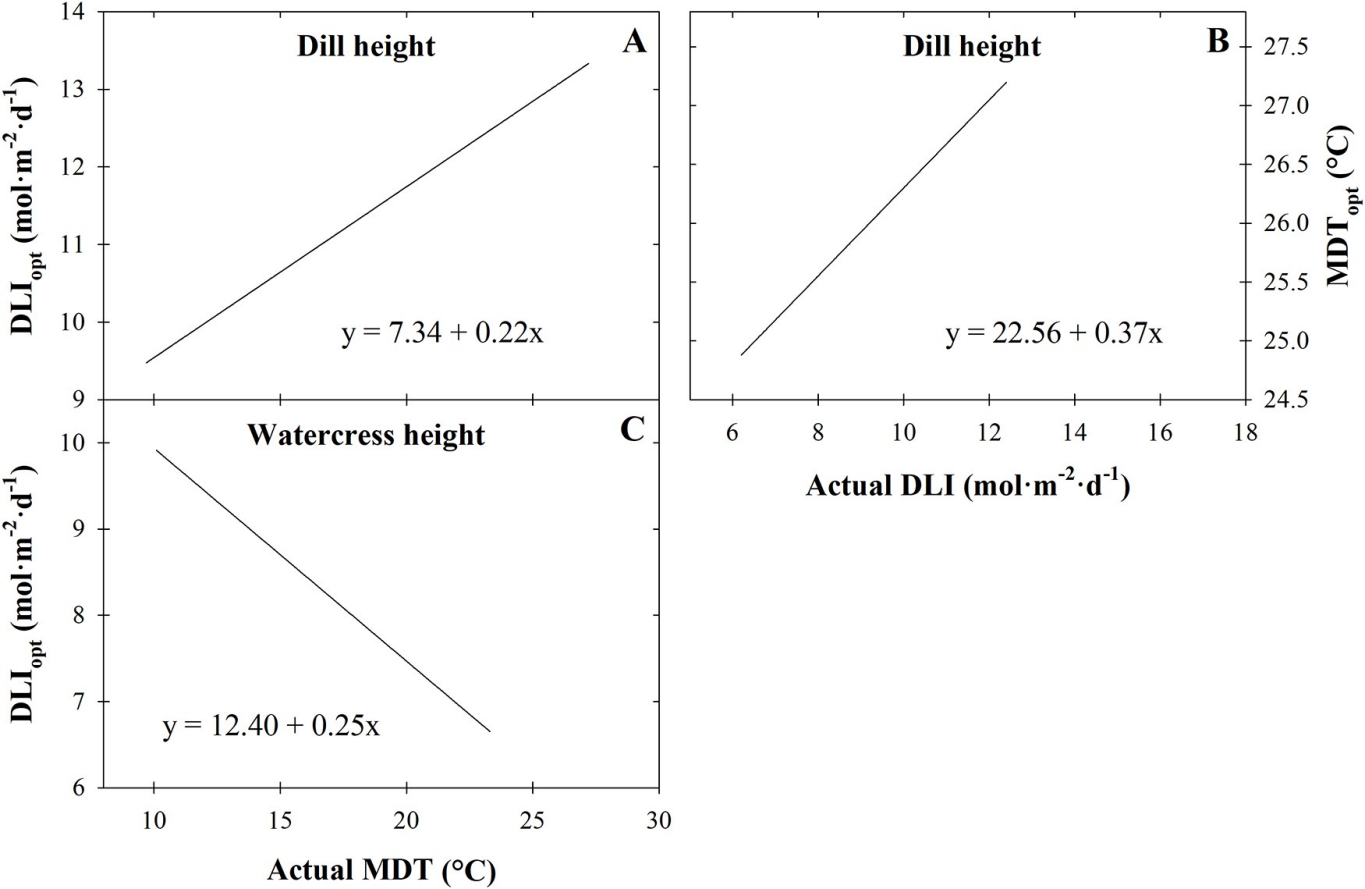
Predicted optimal daily light integral (DLI_opt_; A) and optimal mean daily temperature (MDT_opt_; B) to achieve the tallest dill (*Anethum graveolens*) based on actual MDT and DLI, respectively. Predicted DLI_opt_ (C) to achieve the tallest watercress (*Nasturtium officinale*) based on actual MDT. Equations were generated based on surface regression models (Fig 1C and 1D) with model coefficients reported in Table 1.

To calculate the MDT_opt_ or DLI_opt_ based on DLI or MDT, respectively, supraoptimal MDTs or DLIs must be included in a study. In our study, supraoptimal conditions were observed for dill and watercress height at harvest, but largely not for other parameters measured. Therefore, based on surface regression models (Table 1, Fig 1C and 1D), we calculated the MDT_opt_ for each parameter based on DLI by using *f*_*MDT*opt_ = *a* + 2*c*(*MDT*) + *e*(*DLI*) = 0, and the DLI_opt_ based on MDT using *f*_*DLI*opt_ = *b* + 2*d*(*DLI*) + *e*(*MDT*) = 0 (Fig 4A–4C). Similarly, MDT_opt_ can be calculated based on DLI; the MDT_opt_ for dill height (the MDT to produce the tallest dill, an often undesirable characteristic) is 25 °C when DLI is 7 mol·m^‒2^·d^‒1^, but is 27 °C when DLI is 12 mol·m^‒2^·d^‒1^ (Fig 4B). Though the relationship between the MDT and DLI_opt_ for dill height has a positive slope (Fig 4A), this relationship for watercress height has a negative slope, with the DLI_opt_ decreasing as MDT increases (Fig 4C). Crop height is an important factor to consider when planning for hydroponic culinary herb production. In greenhouses, the distribution of supplemental lighting changes with the distance from the radiation source and plant height should be considered to ensure even radiation distribution for more uniform crops. In indoor vertical production, plant height is even more integral because space between layers should be as close as possible to maximize vertical efficiency. If plants are too tall, leaves can touch light fixtures, leading to non-uniform radiation distribution and potential leaf damage. Based on our models, when DLI is 10 mol·m^‒2^·d^‒1^ and MDT is 15 °C, dill, parsley, and watercress will be 18, 15, and 7 cm tall at harvest (3, 4, and 2 weeks, respectively); whereas if grown at 20 °C, the plants will be 30, 24, and 11 cm tall (Table 1). Additionally, if plants are projected to be too tall, time to harvest can be reduced though yield will be impacted.

Based on our models (Table 1), increasing MDT from 15 to 25 °C will increase fresh mass by 30, 32, and 11 g for dill, parsley, and watercress, respectively, when DLI is 10 mol·m^‒2^·d^‒1^. If the DLI is 15 mol·m^‒2^·d^‒1^, the same increase in temperature will increase parsley and watercress fresh mass similarly, but increase dill fresh mass by 34 g. These equations can be used to more accurately predict yield and crop responses to changing MDT and DLI within the ranges evaluated.

Models were generated using air temperature rather than plant temperature. Though plant responses are due to plant temperature rather than air temperature, air temperature can be used to closely simulate plant temperature, especially when models take both radiation quantity and quality and air temperature into account. Additionally, air temperature is an environmental parameter more commonly monitored and adjusted by producers than leaf temperature [3, 38].

Models generated in this study were based on single plants grown under DLIs ranging from 6.2 to 16.9 mol·m^‒2^·d^‒1^ and MDTs from 9.2 to 27.2 °C for 2, 3, and 4 week production durations for watercress, dill, and parsley, respectively. Predicting growth and development outside of the DLI and MDT ranges used in these models could yield inaccurate predictions. Similarly, trends could be extrapolated (though less accurate) for differing plant densities and production durations; however, these extrapolations would only be qualitative and not quantitative.

## Conclusions

These data allow us to begin modeling plant growth, development, and quality to predict plant responses and conduct cost-benefit analyses. Though technology to precisely manipulate and regulate the growing environment exists, its utility and application is limited when optimal growing conditions are not known. These data will serve as a foundation, allowing growers to calculate and implement the most advantageous growing environment by taking growth, development, and energy costs into account.

## Materials and methods

### Plant production

Seeds of dill ‘Bouquet’, parsley ‘Giant of Italy’, and watercress (Johnny’s Selected Seeds; Winslow, MA) were sown in trays containing stone-wool cubes (2.5 × 2.5 × 4 cm, AO plug; Grodan, Roermond, Netherlands) and placed in a greenhouse. Trays were irrigated overhead daily with reverse-osmosis water supplemented with 12N-1.8P-13.4K water-soluble fertilizer providing (mg·L^–1^) 100 nitrogen, 15 phosphorus, 112 potassium, 58 calcium, 17 magnesium, 2 sulfur, 1.4 iron, 0.5 zinc, 0.4 copper and manganese, and 0.1 boron and molybdenum (RO Hydro FeED; JR Peters, Inc., Allentown, PA) and magnesium sulfate (MgSO_4_) providing (mg·L^–1^) 15 magnesium and 20 sulfur. MDT (~23 °C) was measured by aspirated and shielded 0.13-mm type E thermocouples (Omega Engineering, Norwalk, CT). High-pressure sodium lamps provided a photosynthetic photon flux density (*PPFD*) of ~80 μmol·m^–2^·s^–1^, as measured with a quantum sensor (LI-190R Quantum Sensor; LI-COR Biosciences, Lincoln, NE) every 15 s, and means were logged every hour by a CR-1000 datalogger (Campbell Scientific, Logan, UT) to maintain a 16-h photoperiod and a target DLI of 10 mol·m^‒2^·d^‒1^.

On 25 Feb. 2018 (rep 1) and 1 Mar. 2019 (rep 2), three, four, and five weeks after sowing, watercress, dill, and parsley seedlings, respectively, were transplanted into 18-cm-deep by 0.9-m-wide by 1.8-m-long deep-flow hydroponic systems (270 L Active Aqua premium high-rise flood table; Hydrofarm, Petaluma, CA) in five connected glass-glazed greenhouse compartments with target constant MDTs of 10, 14, 18, 22, or 26 °C. Each greenhouse contained a hydroponic system under 0%, ~30%, and ~50% shade cloth (Solaro 3215 D O FB and Solaro 5220 D O; Ludvig Svensson, Kinna, Sweden) to create target DLIs of 12, 9, or 7 mol·m^‒2^·d^‒1^, respectively.

Hydroponic net pots holding the seedlings were placed in 4-cm-diameter holes, 20-cm-apart, in 4-cm-thick extruded polystyrene floating on the nutrient solution. The nutrient solution consisted of reverse osmosis water supplemented with 12N-1.8P-13.4K water-soluble fertilizer (RO Hydro FeED; JR Peters, Inc.) and MgSO_4_ providing twice the concentrations reported previously. Electrical conductivity (EC) and pH were measured (HI991301 Portable Waterproof pH/EC/TDS Meter; Hanna Instruments, Woonsocket, RI) and adjusted to 1.56 mS·cm^–1^ and 6.0, respectively, by adding fertilizer, reverse osmosis water, potassium bicarbonate, or sulfuric acid. Air pumps (Active Aqua 70 L·min^–1^ commercial air pump; Hydrofarm) and air stones (Active Aqua air stone round 10 cm × 2.5 cm; Hydrofarm) were used to provide dissolved oxygen.

Exhaust fans, evaporative-pad cooling, radiant steam heating, and supplemental lighting were controlled by an environmental control system (Integro 725; Priva North America, Vineland Station, ON, Canada). The photoperiod was 16 h (0600 to 2200 hr), consisting of natural photoperiods (lat. 43° N) and day-extension lighting from high-pressure sodium lamps providing a supplemental *PPFD* of ~150 μmol·m^–2^·s^–1^ when the outdoor *PPFD* was low to maintain target DLIs. Shielded and aspirated 0.13-mm type E thermocouples (Omega Engineering) measured air temperature, infrared thermocouples (OS36-01-T-80F; Omega Engineering) measured leaf temperature of plants grown without shading, thermistors (ST-100; Apogee Instruments, Logan, UT) measured nutrient solution temperature, and quantum sensors (LI-190R Quantum Sensor; LI-COR Biosciences) placed at canopy height recorded *PPFD* in each treatment (reported as DLI, Table 2). Every 15 s, a CR-1000 datalogger (Campbell Scientific) collected environmental data and hourly means were recorded.

**Table 2:** Average daily light integral (DLI; mol·m^‒2^·d^‒1^ ± sd) and mean daily air (MDT), leaf, and nutrient solution temperature over the two (watercress), three (dill), or four (parsley) week growing period for two replications over time.

Rep. and transplant date	DLI	Temperature °C			DLI	Temperature °C			DLI	Temperature °C		
		Air	Leaf	Solution		Air	Leaf	Solution		Air	Leaf	Solution
	Dill ‘Bouquet’ (*Anethum graveolens*)				Parsley ‘Giant of Italy’ (*Petroselinum crispum*)				Watercress (*Nasturtium officinale*)			
1	14.7 ± 3.6	11.4 ± 1.9	16.4 ± 1.1	11.5 ± 1.1	14.7 ± 3.3	10.8 ± 1.8	16.2 ± 1.0	11.2 ± 1.0	13.3 ± 2.5	11.7 ± 2.0	16.4 ± 1.2	11.7 ± 1.2
25 Feb. 2018	11.6 ± 1.8	11.4 ± 1.9	- ^z^	9.4 ± 1.3	11.6 ± 1.8	10.8 ± 1.8	-	9.1 ± 1.2	11.1 ± 1.6	11.7 ± 2.0	-	9.6 ± 1.4
	7.5 ± 1.1	11.4 ± 1.9	-	9.5 ± 1.2	7.6 ± 1.2	10.8 ± 1.8	-	9.0 ± 1.3	7.3 ± 1.1	11.7 ± 2.0	-	9.8 ± 1.2
	14.7 ± 3.3	13.6 ± 1.0	17.3 ± 2.3	14.3 ± 0.9	15.0 ± 3.2	13.3 ± 1.0	17.3 ± 2.3	14.1 ± 0.8	13.4 ± 1.8	13.7 ± 1.1	17.5 ± 2.1	14.3 ± 1.0
	10.5 ± 1.6	13.6 ± 1.0	-	12.6 ± 0.8	10.7 ± 1.7	13.3 ± 1.0	-	12.4 ± 0.8	10.1 ± 1.4	13.7 ± 1.1	-	12.8 ± 0.8
	7.0 ± 1.5	13.6 ± 1.0	-	11.9 ± 0.9	6.9 ± 1.4	13.3 ± 1.0	-	11.6 ± 0.9	6.7 ± 1.4	13.7 ± 1.1	-	12.0 ± 0.9
	15.9 ± 3.6	18.0 ± 0.9	21.3 ± 1.4	16.8 ± 0.6	16.2 ± 3.3	17.9 ± 0.8	20.8 ± 1.4	16.7 ± 0.5	14.3 ± 1.9	18.0 ± 0.9	21.7 ± 1.3	16.9 ± 0.6
	12.2 ± 1.8	18.0 ± 0.9	-	16.5 ± 1.9	12.0 ± 2.0	17.9 ± 0.8	-	16.2 ± 1.6	11.6 ± 1.2	18.0 ± 0.9	-	16.8 ± 2.0
	8.8 ± 1.4	18.0 ± 0.9	-	15.8 ± 0.6	8.6 ± 1.4	17.9 ± 0.8	-	15.7 ± 0.6	8.5 ± 1.2	18.0 ± 0.9	-	16.0 ± 0.7
	12.7 ± 2.0	21.6 ± 0.9	26.1 ± 1.0	19.2 ± 0.9	12.8 ± 1.9	21.3 ± 1.0	25.6 ± 1.2	19.0 ± 0.9	12.1 ± 1.6	21.4 ± 0.9	26.2 ± 1.0	19.2 ± 1.0
	11.3 ± 1.8	21.6 ± 0.9	-	18.8 ± 0.8	10.7 ± 1.9	21.3 ± 1.0	-	18.8 ± 0.8	10.7 ± 1.3	21.4 ± 0.9	-	18.8 ± 0.9
	8.3 ± 1.3	21.6 ± 0.9	-	18.5 ± 1.3	8.5 ± 1.3	21.3 ± 1.0	-	18.5 ± 1.3	8.1 ± 1.1	21.4 ± 0.9	-	18.5 ± 1.4
	13.8 ± 3.4	26.9 ± 0.4	29.1 ± 0.7	23.6 ± 0.7	14.5 ± 3.6	26.9 ± 0.4	29.0 ± 0.6	23.7 ± 0.6	12.2 ± 1.5	26.8 ± 0.4	29.1 ± 0.7	23.6 ± 0.8
	10.6 ± 1.8	26.9 ± 0.4	-	23.6 ± 0.5	10.7 ± 2.0	26.9 ± 0.4	-	23.5 ± 0.4	9.9 ± 1.2	26.8 ± 0.4	-	23.6 ± 0.6
	8.7 ± 1.4	26.9 ± 0.4	-	22.0 ± 0.3	9.1 ± 1.5	26.9 ± 0.4	-	21.9 ± 0.3	8.8 ± 1.4	26.8 ± 0.4	-	22.0 ± 0.3
2	15.6 ± 3.3	9.7 ± 1.1	13.0 ± 1.1	10.3 ± 1.0	17.1 ± 4.2	10.0 ± 1.3	13.2 ± 1.4	10.4 ± 1.0	15.4 ± 3.3	10.1 ± 1.0	13.4 ± 1.0	10.9 ± 0.7
1 Mar. 2019	11.5 ± 2.1	9.7 ± 1.1	-	10.3 ± 0.9	12.6 ± 2.9	10.0 ± 1.3	-	10.3 ± 1.0	11.5 ± 2.2	10.1 ± 1.0	-	10.8 ± 0.6
	8.2 ± 1.6	9.7 ± 1.1	-	-	8.7 ± 1.7	10.0 ± 1.3	-	-	8.3 ± 1.8	10.1 ± 1.0	-	-
	15.3 ± 3.7	13.9 ± 0.5	17.1 ± 0.7	13.8 ± 0.4	17.1 ± 4.7	13.9 ± 0.8	17.2 ± 0.9	13.9 ± 0.6	15.3 ± 4.0	13.9 ± 0.6	17.2 ± 0.8	13.8 ± 0.5
	11.2 ± 2.5	13.9 ± 0.5	-	13.5 ± 0.5	11.6 ± 3.0	13.9 ± 0.8	-	13.6 ± 0.7	11.6 ± 2.4	13.9 ± 0.6	-	13.7 ± 0.5
	8.1 ± 1.7	13.9 ± 0.5	-	13.7 ± 0.1^y^	8.3 ± 1.6	13.9 ± 0.8	-	13.7 ± 0.1^y^	8.3 ± 1.8	13.9 ± 0.6	-	13.7 ± 0.1^y^
	14.1 ± 3.5	18.4 ± 0.5	21.5 ± 0.5	17.3 ± 0.3	16.1 ± 5.0	18.3 ± 0.5	21.5 ± 0.5	17.5 ± 0.4	14.7 ± 3.5	18.5 ± 0.5	21.5 ± 0.4	17.3 ± 0.3
	10.3 ± 2.2	18.4 ± 0.5	-	17.2 ± 0.3	11.2 ± 2.9	18.3 ± 0.5	-	17.3 ± 0.4	10.7 ± 2.2	18.5 ± 0.5	-	17.4 ± 0.2
	8.1 ± 2.2	18.4 ± 0.5	-	15.7 ± 0.4	8.5 ± 3.0	18.3 ± 0.5	-	15.7 ± 0.4	8.7 ± 1.8	18.5 ± 0.5	-	15.8 ± 0.4
	12.9 ± 3.8	22.5 ± 0.4	23.1 ± 0.7	19.9 ± 0.7	14.4 ± 4.6	22.4 ± 0.4	23.5 ± 0.9	20.1 ± 0.8	13.9 ± 3.8	22.4 ± 0.4	22.9 ± 0.7	19.6 ± 0.7
	9.8 ± 2.5	22.5 ± 0.4	-	20.0 ± 0.4	10.4 ± 3.2	22.4 ± 0.4	-	20.1 ± 0.5	10.5 ± 2.3	22.4 ± 0.4	-	19.9 ± 0.5
	6.5 ± 2.4	22.5 ± 0.4	-	19.8 ± 0.4	6.6 ± 2.3	22.4 ± 0.4	-	19.8 ± 0.4	7.3 ± 2.2	22.4 ± 0.4	-	19.7 ± 0.5
	15.0 ± 4.0	27.2 ± 0.3	28.1 ± 0.4	24.1 ± 0.5	16.9 ± 5.1	27.1 ± 0.4	28.2 ± 0.6	24.3 ± 0.5	15.4 ± 4.5	27.2 ± 0.3	28.1 ± 0.4	24.0 ± 0.5
	8.7 ± 2.3	27.2 ± 0.3	-	23.3 ± 0.4	9.8 ± 3.0	27.1 ± 0.4	-	23.5 ± 0.7	9.4 ± 2.4	27.2 ± 0.3	-	23.2 ± 0.5
	6.2 ± 2.3	27.2 ± 0.3	-	23.1 ± 0.9	6.7 ± 2.5	27.1 ± 0.4	-	23.4 ± 1.0	6.9 ± 2.0	27.2 ± 0.3	-	22.8 ± 0.9

^z^ data not collected.

^y^ partial data reported.

### Growth data collection and analysis

The experiment was organized in a split-plot design with each of five MDTs in separate greenhouse sections and three DLI treatments in each section. The experiment was conducted twice over time. At transplant, dill, parsley, and watercress leaf number and fresh and dry mass, and watercress stem length were recorded. Watercress, dill, and parsley plants were harvested when one treatment reached individual marketable size, two, three, or four weeks after transplant. The most recent fully expanded leaf of five watercress and parsley plants in each treatment was dark acclimated for >15 min using manufacturer-supplied clips. Dark-acclimated leaves were exposed to 3,500 μmol·m^–2^·s^–1^ of red radiation (peak wavelength 650 nm) to saturate photosystem II, fluorescence was measured, and F_v_/F_m_ was calculated and reported by a portable chlorophyll fluorescence meter (Handy Plant Efficiency Analyzer (PEA); Hanstech Instruments Ltd. Norfolk, UK). The number of fully expanded dill and parsley leaves, number of watercress branches >2.5 cm, length of the longest watercress branch, plant height from the substrate surface to the tip of the tallest leaf, and fresh mass were recorded for 9 plants per treatment. Tissue was placed in a forced-air oven maintained at 75 °C for at least 3 d, weighed, and dry mass was recorded. DMC was calculated as g dry mass per kg fresh mass. Seedling data were subtracted from harvest data for analysis. Analysis of variance was performed using JMP (version 12.0.1, SAS Institute Inc., Cary, NC); when interactions were not present, data were pooled. Linear, quadratic, and surface regression analyses were conducted using SigmaPlot (version 11.0, Systat Software Inc., San Jose, CA). Equations used to generate predictive models were based on 270 observations for each species.

## Abbreviations

DLI: daily light integral
DLI_opt_: optimal daily light integral
DMC: dry matter concentration
F_v_/F_m_: maximum quantum yield
MDT: mean daily temperature
MDT_opt_: optimal mean daily temperature
PPFD: photosynthetic photon flux density
T_b_: base temperature
T_max_: maximum temperature
T_opt_: optimum temperature
